# Comprehensive Dissection of PDGF-PDGFR Signaling Pathways in PDGFR Genetically Defined Cells

**DOI:** 10.1371/journal.pone.0003794

**Published:** 2008-11-24

**Authors:** Erxi Wu, Nathan Palmer, Ze Tian, Annie P. Moseman, Michal Galdzicki, Xuetao Wang, Bonnie Berger, Hongbing Zhang, Isaac S. Kohane

**Affiliations:** 1 Informatics Program, Children's Hospital Boston, Harvard Medical School, Boston, Massachusetts, United States of America; 2 Division of Health Sciences and Technology, Harvard University and Massachusetts Institute of Technology, Cambridge, Massachusetts, United States of America; 3 Computer Science and Artificial Intelligence Laboratory, Massachusetts Institute of Technology, Cambridge, Massachusetts, United States of America; 4 Department of Mathematics, Massachusetts Institute of Technology, Cambridge, Massachusetts, United States of America; 5 Department of Physiology & Pathophysiology, National Laboratory of Medical Molecular Biology, Institute of Basic Medical Sciences, Chinese Academy of Medical Sciences and Peking Union Medical College, Tsinghua University, Beijing, China; Fred Hutchinson Cancer Research Center, United States of America

## Abstract

Despite the growing understanding of PDGF signaling, studies of PDGF function have encountered two major obstacles: the functional redundancy of PDGFRα and PDGFRβ in vitro and their distinct roles in vivo. Here we used wild-type mouse embryonic fibroblasts (MEF), MEF null for either PDGFRα, β, or both to dissect PDGF-PDGFR signaling pathways. These four PDGFR genetically defined cells provided us a platform to study the relative contributions of the pathways triggered by the two PDGF receptors. They were treated with PDGF-BB and analyzed for differential gene expression, in vitro proliferation and differential response to pharmacological effects. No genes were differentially expressed in the double null cells, suggesting minimal receptor-independent signaling. Protean differentiation and proliferation pathways are commonly regulated by PDGFRα, PDGFRβ and PDGFRα/β while each receptor is also responsible for regulating unique signaling pathways. Furthermore, some signaling is solely modulated through heterodimeric PDGFRα/β.

## Introduction

Platelet-derived growth factor (PDGF) is the principal mitogen in serum for mesenchymal cells and consists of a family of A, B, C, and D polypeptides which promote cell migration, proliferation, and survival by binding to their cognate homo- or heterodimeric tyrosine kinase receptors, PDGFRα and PDGFRβ[1], [2], [3]. Enhanced signaling of PDGF has been implicated in the pathogenesis of atherosclerosis, balloon injury induced restenosis, pulmonary fibrosis, angiogenesis, and tumorigenesis [4].

Tumor growth can be promoted by PDGF via autocrine stimulation of malignant cells, by overexpression or overactivation of PDGFRs, or by PDGF stimulation of angiogenesis within the tumor. Constitutive activation of PDGFRα or PDGFRβ is seen in myeloid malignancies as a consequence of fusion to diverse partner genes, and activating mutations of PDGFRα are seen in gastrointestinal tumors (GISTs). Active PDGFRα was also found in non-small cell lung cancer[*5*]. Autocrine signaling as a consequence of PDGF overexpression has been implicated in the pathogenesis of dermatofibrosarcoma protruberans (DFSP) and overexpression of PDGFRs and/or their ligands has been described in many other solid tumors such as medulloblastomas and malignant gliomas [6], [7]. Therefore, PDGFRs have increasingly become targets for anticancer therapeutics and antirestenosis agents. Two main approaches have been taken toward the inhibition of cancer growth when PDGF-PDGFR signaling is activated: (a) direct targeting of tumor cells in which PDGF signaling is activated, and (b) indirect inhibition of tumors by targeting pericytes to block tumor angiogenesis independently of PDGF activity. A PDGFR inhibitor, imatinib mesylate (Gleevec, STI-571), has benefited patients with myeloid malignancies, GIST and DFSP [8]. PI3K-AKT-mTOR cascade is one of the most frequently deregulated pathway in cancers [9], [10], [11]. Recently we have found that the PDGF receptors are critical for the PI3K/AKT activation and negatively regulated by mTOR. This negative feedback mechanism is important in the prevention of aberrant cell proliferation/growth such as tumor formation and has significant implication in the targeted inhibition of this pathway for cancer treatment [12], [13].

Despite the growing understanding of PDGF signaling, studies of PDGF function have met two obstacles. First, PDGF stimulates a very similar set of cellular responses and signaling events in cultured cells expressing only PDGFRα or PDGFRβ. Because of their functional redundancy or compensation of the receptors with respect to one another *in vitro*, the signaling events of PDGFRα or PDGFRβ cannot be readily analyzed and differentiated. Second, in contrast to *in vitro* studies, PDGFRα and PDGFRβ have dramatically different roles *in vivo*. The mechanism of PDGFR signaling during development is poorly understood because deletion of either the PDGFRα or PDGFRβ leads to early embryonic lethality. While PDGFRβ null embryos are only deficient in smooth muscle cells, particularly vascular smooth muscle cells and pericytes, a large number of different mesenchymal cells are affected in PDGFRα null embryos. The distinct phenotypes of mice lacking either PDGFRα or PDGFRβ suggest that PDGFRs might have unique effectors and/or distinct spatial and temporal expression pattern *in vivo*.

In this study we employed a panel of PDGFR genetically defined cell lines as a platform that allows us to examine the relative contributions of the two receptors to PDGF signaling. We studied the gene expression profile and *in vitro* proliferation assays of the four different genotypes of PDGF receptors: PDGFRα/β double null, PDGFRβ null, PDGFRα null or WT PDGFRα/β in MEF cells. These profiles were then dissected analytically using gene set oriented techniques and complementary data from protein interaction databases. The genes identified in this analysis were then investigated further via protein expression and phosphorylation status analyses. Their functional relevance was then studied.

## Results

### Characterization of PDGFR knockout cell lines

**Figure 1:**
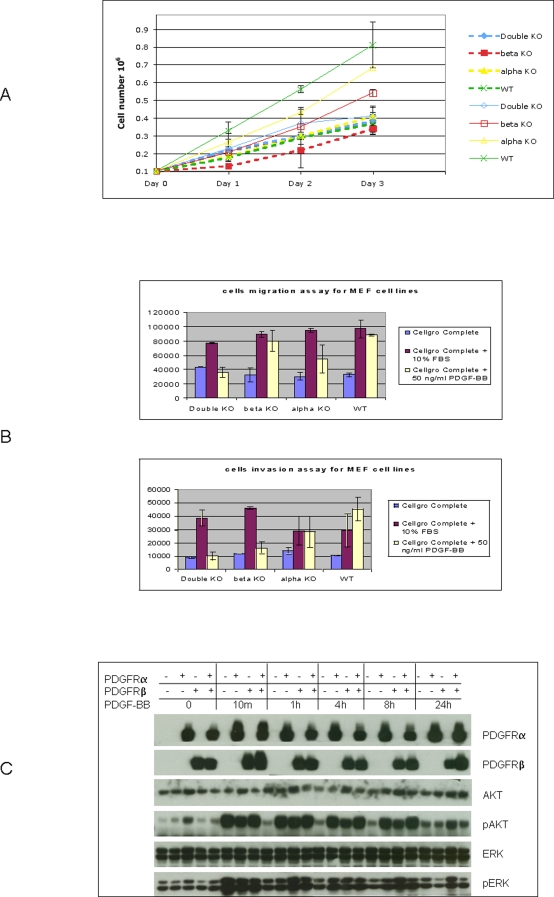
Characterization of four PDGFR genetically defined MEF cell lines. A: PDGF-BB promotes cell proliferation through PDGFR. 1×10^5^ cells were plated at Day 0 and then counted each day for next consecutive three days. Dot lines: cells grown in FBS-free Cellgro Complete medium. Solid lines: cells grown in FBS-free Cellgro Complete medium with PDGF-BB (50 ng/ml). B: PDGFR is required for PDGF mediated cell migration and invasion. Cell migration and invasion were measured by a 24-well chamber-based assay. 2.5×10^4^ cells were seeded in the upper chamber in FBS free Cellgro Complete medium. The lower chamber was filled with Cellgro Complete medium with no FBS, 10% FBS or PDGF-BB (50 ng/ml). After 24 h, the cells in the lower chamber were labeled with Calcein AM and detached. The detached labeled cells were then measured for fluorescence. Upper panel: Migration assay. Lower panel: Invasion assay. C: PDGF-mediated PI3K and MAP kinase signalings are absent in PDGFRα and PDGFRβ double deficient cell lines. Four cell lines were treated with PDGF-BB (50 ng/ml) for 10 min, 1 hour (h), 4 h, 8 h and 24 h. The harvested lysates were immunoblotted for PDGFRα, PDGFRβ, p-ERK (Tyr 204), ERK, p-AKT (Ser473), and AKT. Data shown is one of the representative experiments.

To investigate the role of PDGFRs in cellular proliferation in response to PDGF stimulation, cells were grown in serum free Cellgro COMPLETE™ medium with or without PDGF. In the absence of PDGF, the proliferation rates of all four PDGFR genetically defined cell lines were found to be similar. However, with the addition of PDGF-BB, PDGFRβ null, PDGFRα null and WT cells proliferate faster than the PDGFRα/β double null cells (Figure 1A). In addition, we examined the role that each of the PDGF receptors plays in promoting cell migration and invasion. PDGFRα, PDGFRβ and heterodimeric PDGFRα/β were demonstrated to promote cell migration and also PDGFRβ and heterodimeric PDGFRα/β to enhance cell invasion (Figure 1B).

**Figure 2:**
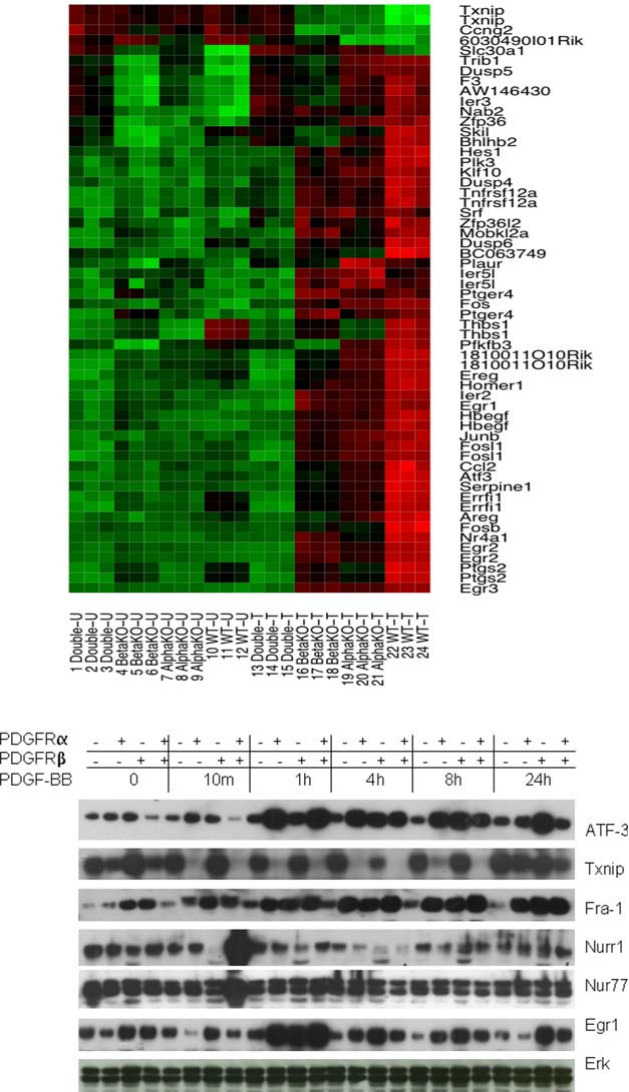
Representative PDGFR dependent differential gene expression in response to PDGF treatment. Upper panel: triplicate sets of each cell line were treated with or without PDGF-BB for 1 h. mRNA expression profiles for the probe sets identified as differentially expressed in the treatment group for all of the cell lines, except for double knockout. Expression levels of the probe sets interrogating the genes that were commonly differentially expressed in the PDGFRβ−/− (beta null), PDGFRα−/− (alpha null), and WT (PDGFRα+/+ PDGFRβ+/+) cell lines are shown in the heatmap. Each column represents a sample, each row a gene. Column labels indicate the cell line and treatment condition (T for samples treated with PDGF-BB, U for untreated samples) for each sample. Each probe set's expression has been independently normalized across the experiments. Bright green shading indicates an expression level below the gene-wise mean, bright red indicates an expression level above the mean, while darker shades indicate expression levels closer to the mean intensity. Lower panel: four cell lines were treated with PDGF-BB (50 ng/ml) for various times and their lysates were immunoblotted for ATF-3, Txnip, Fra-1, Nurr1, Nur77, TF, and EGR1 (PDGF treatment does not influence the total Erk, therefore Erk was regarded as a spotting control in this system, the same for the following experiments in the MEF cell lines with genetically defined PDGFRs) to validate findings from the mRNA expression analysis (upper panel). Expression levels of probe sets interrogating the genes for these proteins are shown in the heatmap above the western blot figure.

**Figure 3:**
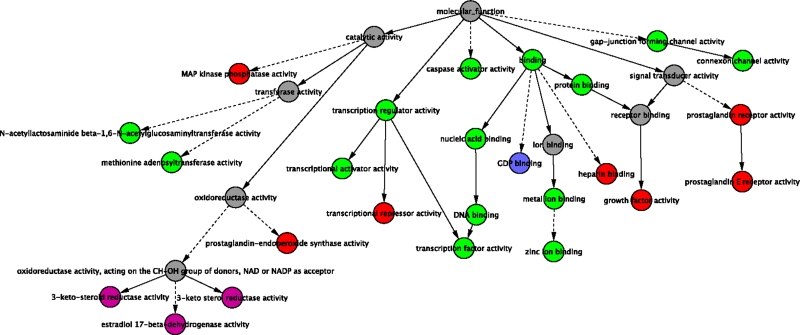
Categories within the Molecular Function GO hierarchy that were overrepresented among the genes that responded to PDGF-BB treatment. Red nodes represent GO terms that were overrepresented among the genes identified as responding to PDGF-BB treatment in the PDGFRα null, PDGFRβ null and WT cell lines. Magenta nodes represent GO terms that were overrepresented among the genes that responded in the PDGFRβ null and WT cell lines, but not in the PDGFRα null cell line. Dark blue nodes represent GO terms that were overrepresented among the genes that responded only in the PDGFRα null cell line. Green nodes represent GO terms that were overrepresented among the genes responding in only the WT cell line. GO categories associated with gray nodes are presented to illustrate context within the GO hierarchy, and were not overrepresented among any of the cell lines. Solid lines represent direct relationships between parent and child nodes in the GO tree, while dotted lines represent long branches of the GO tree containing nodes that were not identified in this analysis.

**Figure 4:**
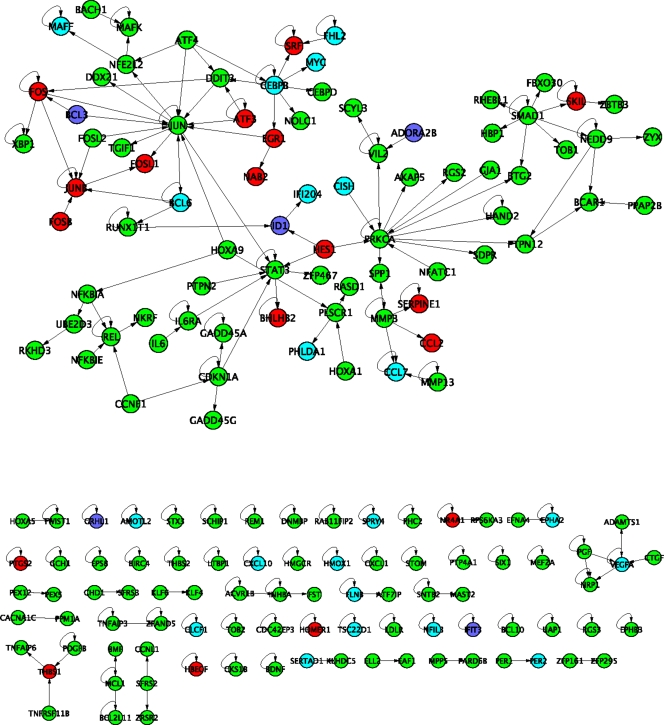
Protein interaction map for differentially expressed genes. The HPRD database was searched for records that reference genes that were differentially expressed in one or more of the PDGFR defined cell lines after stimulation with PDGF-BB ligand. The network shown here represents all of the records in HPRD where both interacting proteins were among the differentially expressed genes. Red nodes represent genes that were differentially expressed in the alpha null, beta null and WT cell lines. Cyan nodes represent genes that were differentially expressed in the alpha null and WT cell lines, but not in the beta null. Dark blue nodes represent genes that were differentially expressed only in the alpha nullcell line. Green nodes represent genes that were differentially expressed only in the WT (heterodimer) cell line.

**Figure 5:**
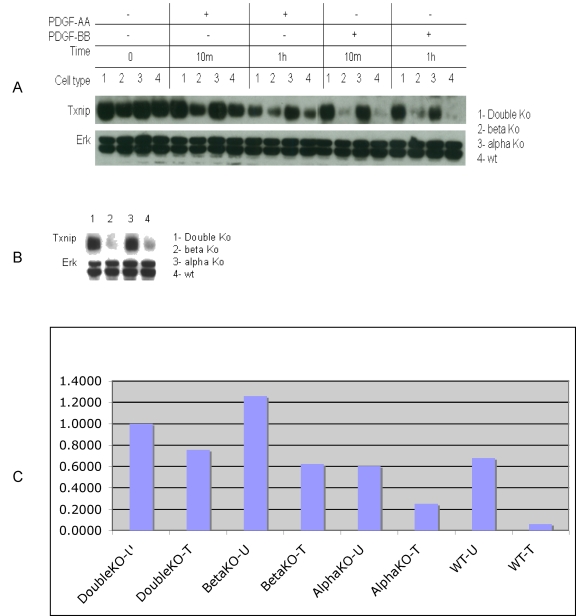
Activation of PDGFRα suppresses Txnip expression. Txnip was downregulated in PDGFRβ null (beta KO) and WT cell lines with PDGF-BB and AA treatments (A) and (C) or with regular medium (10% FBS containing DMEM) (B). A: Immunoblotting for Txnip and ERK in four cell lines treated with PDGF-BB (50 ng/ml) and PDGF-AA (50 ng/ml) for 10 min and 1 h. B: Immunoblotting for Txnip and ERK from the same cells grown in regular medium. C: RNA was extracted from 4 cell lines (double KO, Beta KO, Alpha KO and WT) treated without or with PDGF-BB (50 ng/ml) for 1 h. Txnip gene expression was assessed by reverse transcription-quantitative PCR and normalized using GAPDH as the internal control. The result is one representative of three independent experiments.

**Figure 6:**
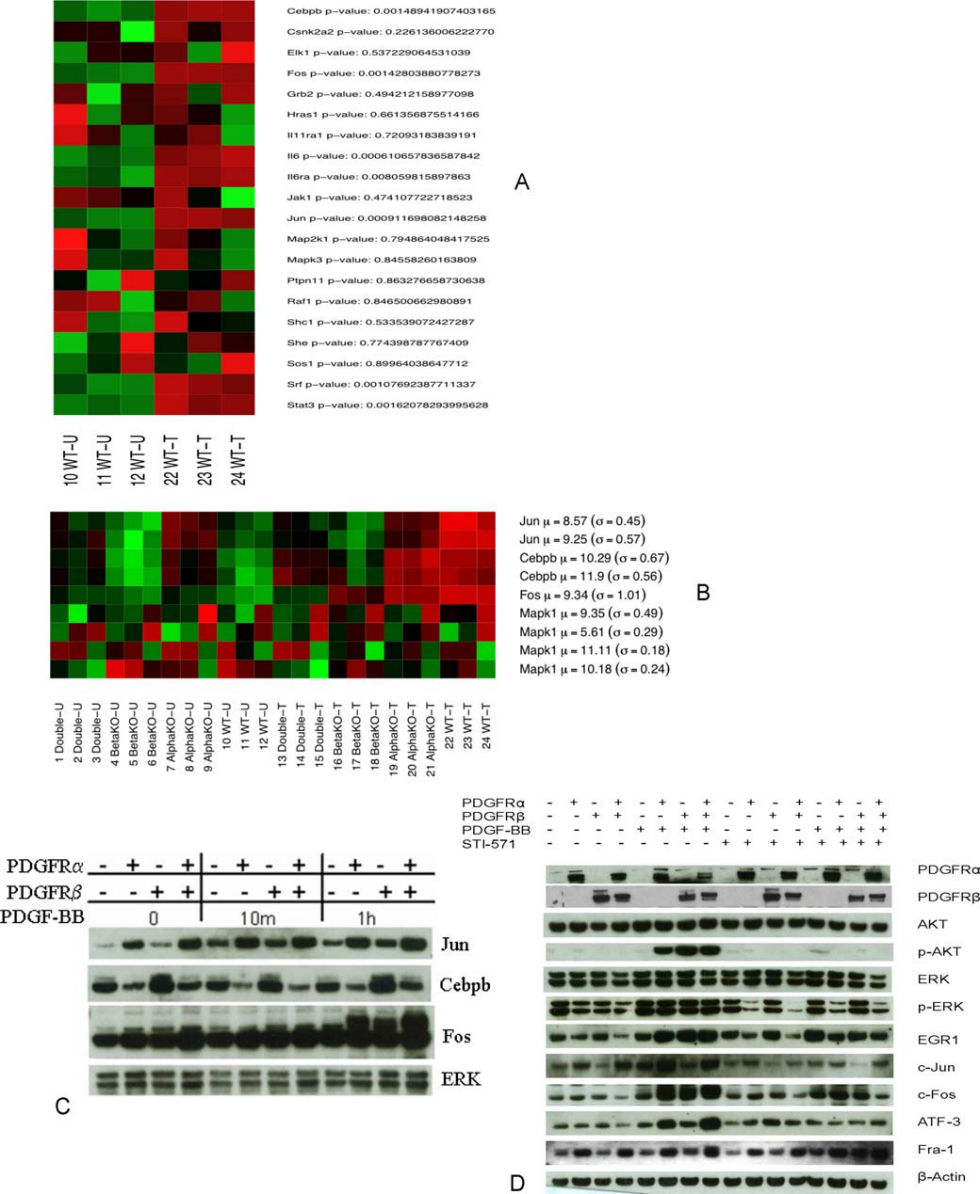
PDGFR regulates IL6 pathway and the signaling pathways modulated by STI-571. A and B: Triplicate sets of each cell line were treated with or without PDGF-BB (50 ng/ml) for 1 h. Expression levels of the genes in the IL6 pathway in the WT cell line are shown in panel A. Expression values are summarized over multiple probe sets for each gene, and a standard Student's T-test p-value indicates the strength of the difference-of-the-means between the treatment groups. In panel B, the expression patterns (across all cell lines) of a few selected genes from the IL6 pathway are shown along with their respective Western blot results in panel C. In both heatmaps, bright green shading indicates an expression level below the gene-wise mean, bright red indicates an expression level above the mean, while darker shades indicate expression levels closer to the mean intensity. The mean and standard deviation for each (log-reduced) gene are shown to the right of each gene name. C: Four cell lines were treated with PDGF-BB (50 ng/ml) for 10 min, 1 h. Cell lysates were immunoblotted for Jun, Cebpb, Fos and ERK (lower panel). D: Four cell lines were pretreated with STI-571 (5 µM) for 90 minutes and then stimulated with PDGF-BB (50 ng/ml) for 1 h. The harvested lysates were immunoblotted for PDGFRα, PDGFRβ, AKT, p-AKT (Ser473), p-ERK (Tyr 204), ERK, EGR1, c-Jun, c-Fos, ATF-3, Fra-1, and β-actin (β-actin as internal control).

Furthermore, PDGF-PDGFR is a known trigger of at least two pathways: the PI3K-AKT and MAPK pathways. To investigate the ability of PDGF to stimulate these pathways in our system, we treated cells with 50 ng/ml of PDGF-BB for various durations. For 10 min post treatment, ERK protein in PDGFRβ null, PDGFRα null, and WT cell lines was extensively phosphorylated, compared with the no treatment by PDGF-BB (Figure 1C). The phosphorylation of AKT protein at various time points showed a similar pattern to ERK phosphorylation and decreased after 24 h. However, in the PDGFRα and PDGFRβ double null cell line, neither ERK nor AKT phosphorylation was increased after the PDGF-BB stimulation.

### Differentially Expressed Genes

To study the role of PDGFRs in PDGF mediated transcription, cells were treated with or without PDGF-BB for 1 h. Analysis of the microarray expression data yielded lists of transcripts that were differentially expressed (responded to PDGF-BB) with high statistical confidence in each of the four cell lines. As described in the Experimental Procedures section, the Significance Analysis of Microarrays (SAM) [14] method was used to identify differentially expressed genes with a false discovery rate <0.05. By comparing these lists, we identified genes that responded to PDGF-BB treatment uniquely in each cell line, and also genes that responded to treatment in multiple cell lines. For example, Figure 2 shows a heatmap describing the transcriptional activity of the genes that were identified in common among the PDGFRβ null, PDGFRα null and WT cell lines (genes such as Txnip, Fos, Egr1, Egr2, Fra-1 (FOSL1), ATF-3, and NR4A1 were all identified as differentially expressed in this particular comparison). The complete list of genes affected by treatment in each cell line can be found in the Supplemental Material (see Table S1 Diff-Genes-PDGF-BB-Treatment.xls).

### Gene Ontology (GO) terms associated with differentially expressed genes

In order to identify functional similarities among the genes that were differentially expressed in one or more of the treatment conditions, we identified GO terms that were statistically overrepresented in each list of genes as described in Materials and Methods. Figure 3 shows the GO Molecular Function terms that were enriched for each cell line. Lists enumerating all of the enriched GO terms for each cell line can be found in the Supplemental Material (see Table S2 GO-Enrichment-PDGF-BB-Treatment.xls). The GO terms characterizing the WT alone (in green) cover transcriptional control and apoptotic programs. Examination of which genes are responsible for this enrichment reveals central “actors” in proliferation, differentiation and apoptosis such as: HES1, BHLHB2, JunB, FOSL1, SRF, SKIL, EGR1, NAB2, FOSB, NR4A1, CCL2 and SERPINE1. The PDGFRα null cell line but not the PDGFRβ null or WT showed differential expression of GDP signaling genes (i.e. required the PDGFRβ isoform, but may be repressed by activity of the PDGFRα isoform). Conversely, the differentially expressed GO sets particular to the PDGFRβ null and WT cell lines (i.e. requiring the PDGFRα isoform) characterize ketosteroid metabolism (colored magenta). Red nodes represent those sets that were found to be differentially expressed in all but the double (α/β) null cell lines, and include the MAP kinase pathway, prostaglandin signaling pathways and several other signaling pathways.

### Gene set (pathway) analysis

In addition to the differential expression analysis, we used sigPathway [15] to identify functional groups of genes (e.g., pathways) that exhibited significantly different behavior between the various conditions. As with the differential expression analysis, we first identified gene sets by comparing the treatment to the non-treatment condition for each of the cell lines. We then compared the pathways identified for each cell line to one another in order to determine which functionality could be attributed most strongly to stimulation of each combination of receptors. The double null data was not included in this analysis because our differential expression analysis indicated that there was no significant change in transcriptional activity that resulted from treatment of the double null cell line. Table 1 lists the gene sets identified as responding to treatment in the various cell lines.

**Table 1:** Pathways identified in each of the PDGFR-defined cell lines

Cell Lines	Source	Gene Set
Wild Type Only	GO:0030182	neuron differentiation
	GO:0042417	dopamine metabolism
	BioCarta	Cadmium induces DNA synthesis and proliferation in macrophages
	BioCarta	IL 6 signaling pathway
	GO:0001942	hair follicle development
	BioCarta	NFAT and Hypertrophy of the heart (Transcription in the broken heart)
	GO:0042133	neurotransmitter metabolism
	GO:0051239	regulation of organismal physiological process
	GO:0008083	growth factor activity
	mousepaths	NFκB Signaling Pathway
	GO:0030282	bone mineralization
	GO:0045664	regulation of neuron differentiation
	GO:0030574	collagen catabolism
	GO:0007566	embryo implantation
	GO:0009888	histogenesis
	GO:0030522	intracellular receptor-mediated signaling pathway
	GO:0030518	steroid hormone receptor signaling pathway
	GO:0045638	negative regulation of myeloid cell differentiation
	GO:0043154	negative regulation of caspase activation
	GO:0001719	inhibition of caspase activation
	GO:0001502	cartilage condensation
	GO:0007565	pregnancy
	GO:0006309	DNA fragmentation during apoptosis
	GO:0006921	disassembly of cell structures during apoptosis
	GO:0030262	apoptotic nuclear changes
Beta Null Only	KEGG	Synthesis_and_degradation_of_ketone_bodies
	BioCarta	SREBP control of lipid synthesis
	GO:0008207	C21-steroid hormone metabolism
	GO:0046912	transferase activity, transferring acyl groups, acyl groups converted into alkyl on transfer
	GO:0016229	steroid dehydrogenase activity
	GO:0003918	DNA topoisomerase (ATP-hydrolyzing) activity
	GO:0000123	histone acetyltransferase complex
	GO:0000777	condensed chromosome kinetochore
	GO:0006700	C21-steroid hormone biosynthesis
	BioCarta	Granzyme A mediated Apoptosis Pathway
	GO:0005694	chromosome
	GO:0050728	negative regulation of inflammatory response
	GO:0000278	mitotic cell cycle
	GO:0030529	ribonucleoprotein complex
	GO:0006281	DNA repair
	GO:0009613	response to pest, pathogen or parasite
	GO:0050877	neurophysiological process
	GO:0043207	response to external biotic stimulus
	BioCarta	The information-processing pathway at the IFN-beta enhancer
	BioCarta	Regulation of MAP Kinase Pathways Through Dual Specificity Phosphatases
	GO:0008217	regulation of blood pressure
	KEGG	Cytokine-cytokine_receptor_interaction
	GO:0006974	response to DNA damage stimulus
	GO:0001584	rhodopsin-like receptor activity
	BioCarta	Transcription Regulation by Methyltransferase of CARM1
	BioCarta	Mechanism of Acetaminophen Activity and Toxicity
	GO:0016070	RNA metabolism
	BioCarta	Platelet Amyloid Precursor Protein Pathway
	GO:0004930	G-protein coupled receptor activity
	GO:0015268	alpha-type channel activity
	GO:0015267	channel or pore class transporter activity
	GO:0004709	MAP kinase kinase kinase activity
	GO:0007186	G-protein coupled receptor protein signaling pathway
Alpha Knockout Only	mousepaths	Th1-Th2-Th3
	GO:0006692	prostanoid metabolism
	GO:0006693	prostaglandin metabolism
	BioCarta	Eicosanoid Metabolism
	GO:0007173	epidermal growth factor receptor signaling pathway
	GO:0001525	angiogenesis
	GO:0048514	blood vessel morphogenesis
	GO:0001508	regulation of action potential
	GO:0045670	regulation of osteoclast differentiation
	GO:0046456	icosanoid biosynthesis
	GO:0006690	icosanoid metabolism
	GO:0030316	osteoclast differentiation
	KEGG	Prostaglandin_and_leukotriene_metabolism
	GO:0030224	monocyte differentiation
	GO:0007205	protein kinase C activation
Beta Knockout and Wild Type	GO:0016126	sterol biosynthesis
	GO:0006695	cholesterol biosynthesis
Alpha Knockout and Wild Type	BioCarta	Neuropeptides VIP and PACAP inhibit the apoptosis of activated T cells
	mousepaths	Ca _ NFAT Signaling Pathways
	mousepaths	Breast Cancer _ Estrogen Receptor Signaling
	mousepaths	Signal Transduction in Cancer
	mousepaths	Cardiovascular Disease
	mousepaths	Signal Transduction PathwayFinder
	mousepaths	Nitric Oxide
	mousepaths	Tumor Metastasis
	mousepaths	Autoimmune and Inflammatory Response
	mousepaths	Angiogenesis
	mousepaths	Endothelial Cell Biology
	GO:0008015	circulation
	GO:0008016	regulation of heart contraction rate
	GO:0042552	myelination
	GO:0042553	cellular nerve ensheathment
	GO:0007272	ionic insulation of neurons by glial cells
	GO:0008366	nerve ensheathment
Alpha Knockout and Beta Knockout	GO:0045765	regulation of angiogenesis
	GO:0046457	prostanoid biosynthesis
	GO:0001516	prostaglandin biosynthesis
	GO:0016525	negative regulation of angiogenesis
	GO:0006955	immune response
	GO:0006952	defense response
	GO:0000279	M phase
	GO:0007067	mitosis
	GO:0000087	M phase of mitotic cell cycle
	GO:0003735	structural constituent of ribosome
	GO:0005840	ribosome
	KEGG	Ribosome
Alpha Knockout, Beta Knockout and Wild Type	mousepaths	cAMP _ Ca Signaling PathwayFinder
	mousepaths	G-Protein Coupled Receptors Signaling PathwayFinder

Several pathways were ranked highly for all three cell lines in this analysis, indicating that these pathways were activated by all of the receptors after PDGF-BB stimulation, including cAMP/Ca signaling and G-protein coupled receptor signaling. In addition, many pathways were highly-ranked in only a strict subset of the cell lines. For example, the IL6 and NFκB signaling pathways were both stimulated in the WT cell line; C21-steroid hormone biosynthesis was stimulated in the PDGFRβ null cell line; angiogenesis and epidermal growth factor receptor signaling pathway were both stimulated in the PDGFRα null cell line.

### All PDGF signaling is PDGFR-dependent

None of the mRNA targets probed by the microarrays exceeded our differential expression threshold in the double null cell line, indicating that transcriptional response to PDGF-BB ligand is mediated entirely through activation of one or both of the PDGF receptors. Pearson correlation coefficients were computed between the mRNA expression fold-change vectors for each cell line (Table 2). This analysis revealed that transcriptional responses to PDGF-BB in the PDGFRα null and PDGFRβ null cells are more similar to the expression response of the WT cells than they are to one another, or the double null cells. The double null cell line's response to PDGF-BB was essentially uncorrelated with that of the other three cell lines.

**Table 2:** Pearson correlation between the vectors of fold change values (all probe sets, treated v.s. untreated conditions) for the four cell lines

Pearson Correlation	Double null	Alpha null	Beta null	WT
**Double null**	1.000	0.0712	−0.034	0.041
**Alpha null**	0.071	1.000	0.719	0.780
**Beta null**	−0.035	0.719	1.000	0.770
**WT**	0.0417	0.780	0.770	1.000

Notice that the responses of both the PDGFRβ knock out cell line and PDGFRα null cell line to PDGF-BB treatment are more similar to that of the WT cell line than any of the others. The response of the double null cell line is essentially uncorrelated with the response of the other three cell lines.

### PDGF-independent PDGFR function

In order to study the function of the two receptors independent of their activation state, we sought genes whose transcription levels changed between the PDGFRα null, PDGFRβ null and WT samples when compared to the untreated double null samples. We then repeated this analysis using the treated samples (i.e., which genes were differentially expressed in the treated PDGFRα null/PDGFRβ null/WT vs. the treated double null cells). We identified those genes whose expression changes were conserved between the untreated and PDGF-BB treated cells. We suggest that these genes are regulated by the presence or absence of a particular receptor, rather than by activation of a receptor through growth factor ligation. Please see Supplemental Material (see Table S3 PDGF-Independent-Gene-Lists.xls) for complete lists of these transcripts.

### Interactome of dissected pathways of PDGFR reveals central processes

We constructed a protein-interaction network based on the annotations present in the HPRD database. We included all interactions where both proteins were the products of genes that were differentially expressed in at least one cell line. Figure 4 shows this network, with each gene labeled by color to indicate the cell lines where it was differentially expressed. Interpretation of this network must be cautious; at the very least because there is significant bias of protein-interaction databases towards those pathways that are better characterized. Nonetheless, we identified significantly enriched pathways (i.e. hypergeometric *p* value, corrected for multiple hypothesis testing, <0.05) among the various combinations of receptor systems. For example, A2B adenosine receptor related processes were identified among the dark blue nodes (see Table S4 GO-Enrichment-for-PPI-Figure.xls in Supplementary Materials for all pathways).

### Protein level validation of microarray results through selected protein level assays of the PDGFRα and PDGFRβ systems

Immunoblotting was used to examine the effect of PDGF-BB treatment at the protein-level for several of the genes identified by the microarray analysis. As per mRNA expression data, Txnip (thioredoxin-interacting protein) was identified as one of the most significantly down-regulated mRNA transcripts. As shown in Figures 2 and 5, protein expression of Txnip is mainly suppressed by PDGFRα, since Txnip is reduced in the presence of PDGFRα and diminished with the activation of PDGFRα by PDGF stimulation. PDGFRβ may have some effects during longer treatment. To confirm this result, we also performed Western blotting with a different ligand (PDGF-AA, 50 ng/ml) to assess the level of Txnip protein expression. The results showed that Txnip was down-regulated in the cell lines containing PDGFRα and heterodimeric PDGFRα/β (Figure 5A). In the regular medium , Txnip was also down regulated in the PDGFRα (PDGFRβ null cell line) and WT cell line (Figure 5B). PDGF is one of main growth factors in FBS and thus is sufficient to suppress Txnip in PDGFRα and WT cell line. Txnip gene expression was further validated by real time-PCR (Figure 5c).

Protein expression of Nurr1 (Nr4a2) and Nur77 (Nr4a1) are altered only in the PDGFR WT cell line post 10 min stimulation of PDGF-BB. This is consistent with the microarray data, where production of mRNA for Nurr1 was induced only in the WT cell line, and production of mRNA for Nur77 was induced most drastically in the WT cell line, and less so in the PDGFRβ null and PDGFRα null cell lines (Figure 2). EGR1 is an early response gene and its protein expression is up-regulated in the PDGFRβ null, PDGFRα null and WT cell lines, but not in the PDGFR double null cell line at 1 h treatment with PDGF-BB. This is also consistent with the microarray data. ATF-3 is up-regulated in the PDGFRβ null, PDGFRα null and WT cell lines, but not in the PDGFR double null cell line, even at 24 h treatment with PDGF-BB. In the PDGFRα null cell line, protein levels of ATF-3 gradually increase at all time points. This is somewhat different from the PDGFRβ null and WT cell lines where ATF-3 protein levels are increased at early time points and gradually decrease thereafter. This indicates that ATF-3 is regulated differently by PDGFRα, PDGFRβ and heterodimeric PDGFRα/β. These results again agree with the microarray analysis (Figure 2).

### Pathway validation by protein level

As described above, we employed the sigPathway method [15] to identify functional groups of genes that exhibited significantly different behavior between the various conditions. We used Western blotting to validate components of one of the pathways, IL6, identified as up-regulated significantly (p<0.05) in the WT cell line in response to PDGF stimulation (Figure 6 A and B). In this IL6 pathway, the genes Fos, Cebpb (NF-IL6), Jun and IL6 were significantly upregulated (p<0.05). As shown in Figure 6C, a few key component genes such as Cebpb (NF-IL6), Fos and Jun in the IL6 pathway were demonstrated as up-regulated by Western blotting. Fos and Jun were up-regulated with PDGF-BB treatment for 1 h while Cebpb (NF-IL6) was little changed. In the absence of PDGF-BB treatment, Jun expression was slightly higher in the PDGFRβ null and WT cell lines than in the others.

### Pharmacological dissection of the PDGF-PDGFR signaling pathways with STI-571

STI-571 (imatinib mesylae, Gleevec, Novartis, Basel, Switzerland) inhibits phosphorylation of both PDGFRα and PDGFRβ and their downstream targets ERK and AKT [16], [17]. In this study we examined the downstream effects of the drug on the PDGF-PDGFR pathway by inhibiting the different isoforms of the receptor. The dosage 5 µM of STI-571 was selected to inhibit PDGFRα and PDGFRβ efficiently [18].

As in Figure 6D, p-AKT was inhibited in PDGFRβ null, PDGFRα null and WT cell lines, while p-ERK was mainly inhibited in PDGFRα containing cells upon the STI-571 treatment. C-Fos was moderately inhibited in PDGFRβ null and PDGFRα null while its expression was strongly inhibited in WT cells by the drug. ATF3 was not inhibited in PDGFRβ null, PDGFRα null and WT cell lines by STI-571 alone, but was inhibited in PDGFRβ null, PDGFRα null and WT cell lines by STI-571 in the presence of both PDGF-BB. Fra-1 (FOSL1) expression did not change significantly in the PDGFRβ null, PDGFRα null and WT cell lines by STI-571 while its expression was increased in the PDGFRβ null, PDGFRα null and WT cell lines in the presence of both PDGF-BB and STI-571. It is notable that PDGF-BB stimulation could not reverse the STI-571 inhibition effect on some genes' expression even though PDGFR-BB moderately increased the expression of C-Fos and Fra-1 as compared to STI-571 inhibition alone.

## Discussion

In this study, we have demonstrated a first “cut” dissection exercise of the PDGFR signaling systems by using the gene expression profile of the four states of PDGF receptors in the PDGFR genetically defined MEF cells and complemented these results with protein-level validation and pharmacological response studies.

We have confirmed some of the genes previously implicated in the PDGF-PDGFR pathway, such as FOS, NR4A1, ZFP36, EGR2, NR4A2, EGR3, FOSB, ATF3, JUN, IER3, ADRB2, DUSP6, MCL1, RGS2, MYC, F3, BHLHB2, GEM, EGR1, LIF and CEBPB [19], [20]. We also identified the involvement of Axud1, MCl1, Tiparp and Txnip in the PDGF-PDGFR system as previously identified by Chen *et al*. [21] using a microarray-coupled gene-trap mutagenesis method. Furthermore we have been able to add more genes to the list such as PTGS2, ERRFI1, JUNB, FOSL1, ERRFI1, EREG, HBEGF, CH25H, HOMER1, PHLDA1, FOS, KLF10, FOSB, SERPINE1, DUSP5, EREG, GEM, HOMER1, HBEGF, TRIB1, CCL2, NFIL3, LBH, IER2, MMP13, GS2, AREG, RSBN1, LIF, TNFRSF11B, CXCL1, NFKBIZ, DUSP4, CCL7, RSBN1, RGS2, IER3, ARL5B, BTG2, ADAMTS1, BTG2, IER5, HES1, RGS2, AXUD1, MMP3, PTGER4 etc.

PDGFRα and PDGFRβ activate many overlapping signaling pathways. All of the receptors activate the same pathways such as cAMP/Ca+ signaling and G-protein coupled receptor signaling after the PDGF-BB stimulation. However, some signaling pathways are exclusively or predominantly activated by one receptor but not the other. Here, we demonstrated that 33 gene sets were activated by PDGFRα only and 15 genes sets by PDGFRβ only. 25 genes sets were specifically activated by PDGFRα/β heterodimers. For example, PDGFRα/β activated components of the NFκB and IL6 signaling pathways, PDGFRα activated C21-steroid hormone biosynthesis; and PDGFRβ activated the angiogenesis and epidermal growth factor receptor signaling pathways. Previous investigations of the pathways regulated by PDGFRs were done one pathway at a time[22]; here we used a bioinformatics approach to comprehensively analyze ,multiple pathways. Nonetheless, the earlier study suggested the Ca++ fluxes pathway is regulated by both PDGFRα and PDGFRβ [22] and angiogenesis is only transduced by PDGFRβ [22], [23]. Our current study is agreement with the previous study (Table 1).

Txnip was identified here to be highly suppressed by PDGFRα. It is identical to VDUP1 (Vitamin D3 up-regulated protein 1) [24], [25]. Txnip/VDUP1 is a known tumor suppressor, cell cycle inhibitor and a factor contributing to P27kip1 stability [26], [27], [28], [29]. Recently, PDGF has been shown to suppress VDUP1 at the mRNA level [30]. We have confirmed that Txnip is down-regulated by PDGF at both the mRNA level and protein level. Furthermore, we identified PDGFRα as the suppressor of Txnip in response to PDGF signaling (Figure 2 and Figure 5).

NR4A1/Nur77 and NR4A2/Nurr1 genes are two members of nuclear hormone receptor family (including Nur77, Nurr1 and Nor-1 or NR4A1-3) [31]. It has been demonstrated that the constitutive expression of Nur77 may induce apoptosis while transient expression does not [31]. With addition of PDGF, Nurr1 and Nur77 protein levels are transiently up-regulated in 10 min, which may protect cells against apoptosis. This occurs only in the presence of PDGFRα with PDGF stimulation. It also has been demonstrated that Nur77 is a survival effecter protein in the context of TNF alpha mediated signaling [31]. The mechanism for Nur77 as a survival effecter protein needs to be further investigated. A very recent study showed that the third member in the nuclear hormone receptor family NR4A orphan nuclear receptor NOR1 is induced by PDGF and mediates vascular smooth muscle cell proliferation [32].This finding suggests that the nuclear hormone receptor family are therapeutic targets for some diseases in which PDGFRs are overexpressed.

These results are illustrative of the combinatorial richness of PDGF receptor/ligand-mediated signaling. Our results begin to reveal the downstream interplay of the signaling events brought about by the activation of each of the two receptors, indicating the biological effect of receptor/ligand specificity. Furthermore, in this study, we have demonstrated that transcriptional response to PDGF-BB ligand is mediated entirely through activation of one or both of its receptors and suggest that PDGF ligand, PDGF-BB in this study, does not bind any other receptors.

Similar to the stimulation of PDGF, the responsiveness of PDGF receptors to pharmacological inhibition is also complex. While STI-571 inhibits AKT activation through either PDGFRα or PDGFRβ, it blocks ERK activation mainly through PDGFRα (Figure 6). Therefore, this PDGFR platform may help us to further understand the molecular mechanism of therapeutic inhibition on PDGF-PDGFR signaling and identify additional critical molecular targets for the intervention of cancer and other diseases.

The studies presented here are prone to many well-known limitations, such as the noisiness of expression microarrays, the frequent lack of concordance of gene and protein expression, and the post-translational signaling systems that are at most only faintly echoed in gene expression levels. We have attempted to minimize these limitations by employing rigorous statistical techniques, focusing on pathways as much as on individual genes, using protein interaction data to corroborate co-expression findings and using selected protein measurements, including previously implicated post-translational modifications.

In summary, we have taken advantage of the experimental platform presented by PDGFR double null cells, PDGFRα, PDGFRβ and PDGFR WT cell lines (where dimers PDGFRα/α, PDGFRβ/β, PDGFRα/β co-exist) and used a bioinformatics approach to dissect the gene sets/pathways that are controlled by two PDGFR isoforms with PDGF-BB ligand stimulation. Our study also provides a reproducible approach to the dissection of the contributions of a heteromeric receptor signaling system. While minimal PDGF receptor-independent signaling was found, we identified the signals commonly regulated by PDGFRα, PDGFRβ and PDGFRα/β, specifically triggered by each of the two PDGF receptors as well as the heterodimeric PDGFRα/β.

## Materials and Methods

### Cells and viruses

PDGFRα/β double null (double KO) (α−/−β−/−), PDGFR beta null (PDGFRα/β double null cells infected with PDGFRα expressing retro-viruses), and PDGFR alpha null (PDGFRα/β double null cells infected with PDGFRβ expressing retro-viruses) MEFs were gifts from Dr. Andrius Kazlauskas (Schepens Eye Research Institute) [33]. Wild-type PDGFRα and PDGFRβ MEFs [PDGFRα+/+ β+/+] were generated as follows. Human PDGFRβ cDNA was amplified from hPDGFRβ in pEF6 (Invitrogen, Carlsbad, CA) with primers (all primer sequences available upon request) using proof-reading Pfu polymerase (Stratagene, La Jolla, CA). The PCR products were digested with Not I and Cla I (NEB Biolab, Ipswich, MA) and inserted into a retroviral vector pIRES-hygromycin [34]. The plasmids were transfected with Lipofectamine 2000 (Invitrogen, Carlsbad, CA) into the retroviral packaging cell line PT67 (Clontech, Palo Alto, CA). Filtered medium containing viruses carrying PDGFRβ was used to infect PDGFRα+/+ β−/− MEF. Infected cells were then selected with 100 µg/ml hygromycin B (Clontech, Palo Alto, CA). All cells were cultured in DMEM with or without 10% FBS in 5% CO_2_ at 37°C.

### DNA microarray hybridization

Total RNA was extracted using the RNeasy Mini kit (Qiagen, Valencia, CA) from cells that were treated or untreated with or without 50 ng/ml PDGF-BB for 1 h. Five µg RNA of each sample was used for double-stranded cDNA synthesis. *In vitro* transcription was carried out using the Enzo BioArray High Yield RNA Transcript Labeling Kit (Enzo, New York, NY). The labeled cRNA target (20 µg each) was then fragmented into 35–200 base pair fragments and hybridized to each Affymetrix GeneChip® Mouse Genome 430 2.0 array according to Affymetrix Eukaryotic Target Hybridization Protocol. Following a 16-h incubation the microarrays were washed and scanned using the EukGE-WS2v4 fluidics protocol.

### Differential expression analysis

Microarray data were normalized, background corrected and summarized using the robust multi-array average (RMA2) method [35]. We identified individual genes that exhibited statistically significant differential expression as a result of PDGF treatment in each null experiment (i.e., genes induced or repressed in the α−/− β−/−, α+/+ β−/−, α−/−β+/+, α+/+ β+/+ cells when treated with PDGF-BB) using SAM method [14]. In each comparison we selected a value for SAM's Δ parameter to specify a median false discovery rate less than 0.05, and we consider the genes reported at this threshold to be differentially expressed between the treatment and non-treatment conditions.

### Gene set analysis

To identify functional groups of genes from each cell line with RNA expression profiles that were affected by PDGF-BB treatment, we used Tian et al's [15] sigPathway method Gene set annotations were assembled from Gene Ontology [36], KEGG [37], BioCarta (biocarta.com), BioCyc [38] and custom data. This method identifies significantly enriched gene sets by testing two related null hypotheses: 1) The pattern of expression for the genes in a particular gene set is the same as all the other genes, and 2) the gene set does not contain genes with expression profiles that are correlated with treatment. We picked out gene sets that were distinguished by both statistics by ranking the sets by the sum of the ranks of their two statistics. All of the gene sets presented here had a false discovery rate below 0.05 for at least one statistic.

We identified statistically over-represented Gene Ontology (GO) terms for the lists of differentially expressed genes by computing a hypergeometric *p*-value for each GO annotation associated with a given gene list. The resulting *p*-values were corrected for multiple hypothesis testing using the FRD method [39]. An FDR below 0.05 was regarded as indicating significant over-representation.

### Comparison of genes lists

Once lists of genes and pathways whose mRNA expression responded to PDGF-BB treatment were assembled by the above methods, we compared the lists to see which genes had responded to treatment uniquely in one of the nulls or wild type, or common to two or more of the null and wild type conditions.

### Correlation analysis

In order to evaluate how similar each of the four cell lines' transcriptional responses to PDGF-BB ligand stimulation were to one another, Pearson correlation coefficients were computed between all pairs of cell lines. The SAM software package for R was used to compute fold change values (PDGF-BB treated expression values vs. untreated expression values) for all genes present in the normalized microarray data, for each cell line. Thus, four vectors of fold change estimates were obtained (one for double null, one for alpha null, one beta null and one for wild type) representing each cell line's response to ligand stimulation. Pearson correlations between each pair of these vectors were computed using R.

### Western blot analysis

The cells were starved for two days, then left unstimulated, or simulated with 50 ng/ml PDGF-BB (Sigma-Aldrich, Saint Louis, MO) in six well plates in serum-free medium for specific time points. Cell lysates were prepared and proteins were separated by electrophoresis. The electroblotted nitrocellulose membranes were probed with antibodies.

Antibodies were obtained from: PDGFR**α** (C-20), ERK (K-23), p-ERK (E-4), Fra-1 (R-20), Nurr1 (M-196), Nur77 (M-210),TF (I-20), Egr3 (C-24), ATF-3 (C-19), c-Fos (4), Cebpb (H-7) (Santa Cruz Biotech, CA); PDGFRβ **(**Upstate, Temecula, CA**);** p-AKT (Ser473), AKT, EGR1 and c-jun (Cell Signaling Technology, Beverly, MA); Txnip (MBL International, Woburn, MA). Secondary antibodies were anti-mouse IgG, anti-rabbit IgG and anti-goat IgG HRP (Santa Cruz Biotech, CA).

### Cell proliferation, migration and invasion assays

Cells were cultured in Cellgro COMPLETE™ with L-glutamine & phenol red (40-101-CV, Mediatech, Herndon, VA) in the presence or absence of growth/proliferation factor. Cell numbers were counted every day in triplicates using Trypan Blue dye exclusion method for cell viability (Vi-Cell, Beckman Coulter, Fullerton, CA). Migration and invasion assays were performed using 24-well chamber-based plates (BD Biosciences, Bedford, MA). FBS or PDGF-BB was used as chemo-attractants.

### Reverse transcription-quantitative PCR

One µg of RNA from each sample was reverse transcribed using iScript cDNA Synthesis Kit (Bio-Rad Laboratories, Hercules, CA) according to the instructions of the manufacturer. The original cDNA reaction mixture was diluted to one-tenth of the reaction volume. 2 µl of the diluted cDNA was used as the template in the quantitative PCR reaction. Amplification was done using iQ SYBR Green Supermix on an iCycler (Bio-Rad, Hercules, CA) (primer sequences for Txnip and GAPGH available upon request).

### Statistical analysis

Hypergeometric *p*-values were used to evaluate GO term enrichment. The raw *p*-values were corrected for multiple hypothesis testing using the FRD method [39] to compute *q*-values. Any *q*-value below 0.05 was considered significant.

## Supporting Information

Table S1 Diff-Genes-PDGF-BB-Treatment
(0.06 MB XLS)

Table S2 GO-Enrichment-PDGF-BB-Treatment
(0.06 MB XLS)

Table S3 PDGF-Independent-Gene-Lists
(0.08 MB XLS)

Table S4 GO-Enrichment-for-PPI-Figure
(0.12 MB XLS)
